# Supplementary material for: First Molecular Identification of Caligus clemensi on Cultured Crimson Snapper Lutjanus erythropterus on Jerejak Island, Penang, Peninsular Malaysia
Source: Pathogens. 2022 Jan 29;11(2):188. doi: 10.3390/pathogens11020188 (PMC8879188; doi:10.3390/pathogens11020188)
Supplement: Supplementary file 1 [file pathogens-11-00188-s001.zip › pathogens-1508173-supplementary.pdf]

Supplementary Table S1: Measurements of some body parts of *C. clemensi* on cultured Crimson Snapper, *Lutjanus erythropterus* at Jerejak Island, Penang, Peninsular Malaysia.

S/N	Total Body Length	Cephalothorax Shield	Fourth Leg	Genital Complex	Abdomen Somite
C. clemensi 1	3.9–4.1 mm	Length: 2.5 mm; width: 1.9	0.2 mm	0.9–1.0 mm	0.7–1.0 mm
C. clemensi 2	3.9 mm	Length: 2.5 mm; width: 1.9 mm	0.2 mm	0.9 mm	0.7 mm
C. clemensi 3	4.0 mm	Length: 2.6 mm; width: 1.9 mm	0.2 mm	0.9 mm	0.7 mm
C. clemensi 4	4.0 mm	Length: 2.6 mm; width: 1.9 mm	0.2 mm	0.9 mm	0.7 mm
C. clemensi 5	4.0 mm	Length: 2.6 mm; width: 1.9 mm	0.2 mm	0.9 mm	0.7 mm
C. clemensi 6	4.1 mm	Length: 2.7 mm; width: 2.0 mm	0.2 mm	0.9 mm	0.7 mm
C. clemensi 7	4.1 mm	Length: 2.7 mm; width: 2.0 mm	0.2 mm	0.9 mm	0.8 mm
C. clemensi 8	4.0 mm	Length: 2.6 mm; width: 1.9 mm	0.2 mm	0.9 mm	0.8 mm
C. clemensi 9	4.0 mm	Length: 2.6 mm; width: 1.9 mm	0.2 mm	0.9 mm	0.8 mm
C. clemensi 10	4.0 mm	Length: 2.6 mm; width: 1.9 mm	0.2 mm	0.9 mm	0.8 mm
C. clemensi 11	4.0 mm	Length: 2.6 mm; width: 1.9 mm	0.2 mm	0.9 mm	0.8 mm
C. clemensi 12	4.0 mm	Length: 2.6 mm; width: 1.9 mm	0.3 mm	0.9 mm	0.8 mm
C. clemensi 13	3.9 mm	Length: 2.5 mm; width: 1.9 mm	0.3 mm	1.0 mm	0.9 mm
C. clemensi 14	3.9 mm	Length: 2.5 mm; width: 1.9 mm	0.3 mm	1.0 mm	0.9 mm
C. clemensi 15	3.9 mm	Length: 2.5 mm; width: 1.9 mm	0.3 mm	1.0 mm	0.9 mm
C. clemensi 16	3.9 mm	Length: 2.5 mm; width: 1.9 mm	0.3 mm	1.0 mm	0.9 mm
C. clemensi 17	3.9 mm	Length: 2.5 mm; width: 1.9 mm	0.3 mm	1.0 mm	0.9 mm
C. clemensi 18	3.9 mm	Length: 2.5 mm; width: 1.9 mm	0.3 mm	1.0 mm	1.0 mm
C. clemensi 19	3.9 mm	Length: 2.5 mm; width: 1.9 mm	0.3 mm	1.0 mm	1.0 mm
C. clemensi 20	3.9 mm	Length: 2.5 mm; width: 1.9 mm	0.3 mm	1.0 mm	1.0 mm
